# Anti-gingipain antibodies and ACE1 activity in rheumatoid arthritis: associations with disease activity and cardiovascular comorbidity in a cross-sectional study

**DOI:** 10.1007/s00296-026-06272-4

**Published:** 2026-08-25

**Authors:** Marta Kaminska, Maja Chrobak, Lucas Sathler Alves Silva, Yasmin Gurtler Pinheiro de Oliveira, Ewa Bielecka, Arthur Dalmaso Pinto, Ruben Horst Duque, Jose Geraldo Mill, Valeria Valim, Piotr Mydel

**Affiliations:** 1 Broegelmann Research Laboratory, Department of Clinical Science, Faculty of Medicine, University of Bergen, Bergen, Norway; 2 Department of Microbiology, Faculty of Biochemistry, Biophysics and Biotechnology, Jagiellonian University, Kraków, Poland; 3 Department of Rheumatology, University Hospital Cassiano Antônio Moraes – Federal University of Espírito Santo (HUCAM-UFES), Vitória, ES Brazil; 4 Małopolska Centre of Biotechnology, Jagiellonian University, Kraków, Poland; 5 Department of Physiological Sciences, Health Sciences Center (CCS), Federal University of Espírito Santo (UFES), Vitória, ES Brazil; 6 Department of Medicine and Postgraduate Program in Public Health (PPGSC), University Hospital Cassiano Antônio Moraes – Federal University of Espírito Santo (HUCAM-UFES), Vitória, ES Brazil

**Keywords:** Arthritis, Rheumatoid, *Porphyromonas gingivalis*, Gingipain cysteine endopeptidases, Antibodies, bacterial, Peptidyl-Dipeptidase A, Protein-arginine deiminase Type 4, Anti-citrullinated protein antibodies, Biomarkers, Cardiovascular diseases, Cross-sectional studies

## Abstract

We assessed cross-sectional relationships of rheumatoid arthritis (RA) disease activity and cardio-metabolic comorbidities with serum peptidylarginine deiminase 4 (PAD4)-potentiating activity, domain-resolved angiotensin I converting enzyme (ACE1) enzymatic activity, and *Porphyromonas gingivalis*-specific antibodies. 177 RA patients and 147 healthy controls (HC) were included in the study. Clinical profiling included DAS28, CRP/ESR, treatments, and comorbidities. PAD4-potentiating activity, ACE1 activities, and anti-gingipain antibodies were measured using standardized in-house assays. Associations with RA status, treatments, and outcomes were analysed using covariate-adjusted models; discrimination for selected comorbidities was explored with ROC analyses. PAD4-potentiating activity, anti-Kgp/anti-RgpB and ACE1 activities did not differ between RA and controls. In RA, anti-Kgp showed a weak correlation with DAS28 (Rs=0.17, p=0.033) and was lower with biologic disease-modifying antirheumatic drugs (DMARDs); ACE1 activity was lower among synthetic-DMARDs users across all three substrates (all p≈0.01–0.04). None of the biomarkers showed independent associations with DAS28 in adjusted models. Overall discrimination for cardio-metabolic outcomes was limited; small, predefined strata showed exploratory signals, but low event counts and multiple comparisons constrain inference. In this cohort, between-group differences were limited overall. However, two exploratory within-RA signals emerged: anti-Kgp levels were weakly associated with disease activity and therapy, and lower ACE1 activity was associated with synthetic DMARDs use. Together, anti-Kgp and ACE1 (N-domain) may reflect mucosal-immune and vascular-remodeling axes that could relate to comorbidity patterns and warrant prospective, pre-specified validation.

**Supplementary Information:**

The online version contains supplementary material available at https://doi.org/10.1007/s00296-026-06272-4.

## Introduction

The incorporation of anti-citrullinated protein antibodies (ACPA) into the 2010 ACR/EULAR criteria underscored citrullination-linked autoimmunity as a hallmark of RA [1]. Unfortunately, ACPA negativity is common and clinical trajectories vary substantially even among ACPA-positive patients, indicating that autoantibody status alone does not fully capture underlying biology [2]. This has directed attention to the enzymes that generate citrullinated epitopes: peptidylarginine deiminases (PADs), with PAD4 strongly implicated in RA pathogenesis [3, 4]. PAD enzymes contribute to physiological processes (e.g., epithelial differentiation or neutrophil extracellular trap formation) [5–7], but dysregulated PAD activity has been linked to RA and other inflammatory states, including cardiovascular and neurodegenerative diseases [8–10]. Aberrant PAD activity may facilitate breach of tolerance and ACPA generation [11]. A persistent mechanistic question is whether RA autoimmunity is driven by immune responses to a restricted set of citrullinated peptides or reflects broader dysregulation of citrullination. This question intersects with epidemiologic and mechanistic links between RA and periodontitis [12]. *Porphyromonas gingivalis* expresses PAD-like activity and potent proteases (RgpB and Kgp) that can reshape the host proteome and inflammatory milieu, potentially promoting citrullination and neoepitope formation locally and systemically [13, 14]. Transient bacteremia after mucosal injury has been proposed as one route by which oral inflammation could contribute to systemic autoimmunity and ACPA development [15, 16].

 Previously, we developed a fluorescent peptide–based enzymatic assay for recombinant PAD4 activity [17] and showed that serum from newly diagnosed, DMARD-naïve RA patients potentiates PAD4 activity relative to matched healthy controls, supporting serum-mediated modulation of PAD4 as a candidate biomarker of RA-associated immune activity [18].

In parallel, angiotensin-converting enzyme 1 (ACE1) – a key regulator of vascular tone and inflammatory homeostasis – represents a mechanistically distinct but clinically relevant axis, given domain-specific catalytic properties [19–21] and links to endothelial dysfunction and cardiovascular risk [22]. Because RA therapies and common cardiometabolic co-medications can influence inflammatory and vascular pathways, medication context is essential for interpreting PAD4 and ACE1 activity readouts [18].

In this cross-sectional study of a rheumatoid arthritis cohort with healthy controls, we examined whether serum antibodies to *Porphyromonas gingivalis* gingipains, PAD4 activity and ACE1 activity are related to disease activity and cardiovascular comorbidity, with the aim of generating hypotheses for prospective validation.

## Methods

This cross-sectional study is reported in accordance with the STROBE (Strengthening the Reporting of Observational Studies in Epidemiology) guidance for cross-sectional studies, and the completed checklist is provided as Supplementary File S1. The study population comprised the available Risk RA cohort, and no formal a priori sample-size calculation was performed. ACPA status was obtained from the participants’ existing clinical records and was therefore available for a subset of patients (*n* = 85).

### Sample collection and processing

Blood samples were collected through venipuncture at the cubital region, performed by an experienced professional. Ten milliliters of blood were collected in a tube with a separator gel. The serum obtained from centrifuging the samples (1,300 g, for 15 min) was aliquoted and stored at −80 °C until further analysis.

### ACE1 activity

Serum ACE1 activity was assessed using three fluorogenic substrates: Abz-FRK(Dnp)P, Abz-SDK(Dnp)P, and Abz-LFK(Dnp) (Bachem and SigmaAldrich), which are specific for both, N- or C-domains of ACE1, respectively. Five microliters of serum were incubated in PBS supplemented with 10 µM ZnCl₂ at 37 °C, with parallel negative controls including 4.5 µM lisinopril. Substrates were added at a final concentration of 30 µM, and fluorescence was measured every 50 s over 50 min (excitation 320 nm, emission 420 nm) using a microplate reader. Enzymatic activity was expressed as substrate cleavage velocity after subtraction of background signal from the lisinopril-inhibited samples.

### PAD4-activating potential

Serum PAD4-activating potential was determined using a previously established in-house assay [18]. Briefly, 20 µl of serum were incubated with 1 mU recombinant PAD4, 1 mM Dansyl-Gly-Arg substrate, 2 mM o-phenanthroline, and 10 mM DTT in 100 mM Tris buffer (pH 7.5), with positive PAD4 activity control containing 10 mM CaCl₂ (final reaction volume: 30 µl). After 60 min at 37 °C, reactions were terminated with 5% trichloroacetic acid. After protein precipitation and acidification with trifluoroacetic acid, samples were centrifuged, and supernatants were analyzed by HPLC (AERIS Peptide XB-C18 column, Phenomenex). Substrate and citrullinated products were detected at 330/530 nm (excitation/emission). PAD4 activity potentiation was quantified from product peak area and normalized to a reference serum sample included in each analytical run.

### Anti-RgpB and anti-Kgp antibodies

MaxiSorp plates were coated overnight with recombinant RgpB or full-length Kgp protein (2.8 µg/ml) in carbonate buffer, blocked with 2% BSA, and incubated with serum samples diluted 1:400 in RIA buffer (1% BSA, 350 mM NaCl, 10 mM Tris, 1% Triton X-100, 0.1% SDS). After washing, horseradish peroxidase–conjugated goat anti-human IgG (diluted 1:10,000) were added to the wells, and then detected with tetramethylbenzidine substrate. Reactions were stopped with 1 N H_2_SO_4_, absorbance at 450 nm was measured (correction at 570 nm), and antibody levels expressed in arbitrary units (AU).

### Data analysis

All biomarker data were normalized to a reference serum sample included in each experimental batch. Analyses were performed in R (v4.4.3). Continuous and categorical variables were predefined, and raw data were cleaned for consistency. Demographic, clinical, and serological variables were summarized descriptively. Baseline characteristics were compared between RA patients and HC using Student’s t test or Mann–Whitney U test for continuous variables, based on normality assessed by the Shapiro–Wilk test, and χ² or Fisher’s exact test for categorical variables. Data are presented as mean ± SD, median (IQR), or n (%) as appropriate.

Associations between experimental variables (PAD4 activity, ACE1 substrate velocities [FRKP, LFK, SDKP], heparin activity, anti-RgpB and anti-Kgp antibodies, RF titre, Waaler–Rose, latex agglutination, and ACPA) and clinical outcomes (DAS28, CRP, ESR) were initially examined using univariable analyses. Primary analyses compared RA and HC groups (PAD4 activity, ACE1 activity, anti-gingipain antibodies) and evaluated within-RA associations with disease activity and DMARD exposure. Correlations between continuous variables were assessed using Spearman’s test, adjusted for age, sex, ethnicity, smoking status, and disease duration.

Continuous outcomes (DAS28, CRP, ESR) were analysed using linear regression, and binary outcomes (cardiometabolic disease, periodontal disease, and autoantibody positivity) using logistic regression. Variables identified in univariable screening were entered into multivariable models. Independent biomarker contributions were evaluated using a systematic ablation approach, fitting all predictor subsets adjusted for predefined covariates (age, sex, ethnicity, smoking, DMARDs, statin use). Model performance was assessed using AIC, BIC, adjusted R², and for binary outcomes, AUC, Brier score, and calibration metrics. Results are reported as regression coefficients or odds ratios with 95% confidence intervals. All tests were two-sided (*p* < 0.05). Missing data were handled by complete-case analysis. These analyses were exploratory and hypothesis-generating. p-values are reported without formal correction for multiple testing and are interpreted as nominal, with emphasis on effect sizes and confidence intervals rather than significance thresholds; results from small subgroups are interpreted with particular caution.

## Results

### Cohort characteristics

A total of 177 patients with RA and 147 HC were included in the study. The RA cohort comprised 164 females and 13 males, with a mean age of 56.84 ± 10.52 years. RA patients had a mean disease duration of 11.3 ± 8.7 years, calculated from the reported time since diagnosis. Self-reported ethnicity in RA group was predominantly Mixed/Brown and White. HC were frequency-matched to the patient cohort by sex and age with a mean age of 43.09 ± 13.05 years. Most RA patients were receiving DMARDs at the time of sample collection, with 85 receiving biologic DMARDs and 144 on synthetic DMARDs.

### Comparison of biochemical markers and antibody levels between RA patients and HC

**Fig. 1:**
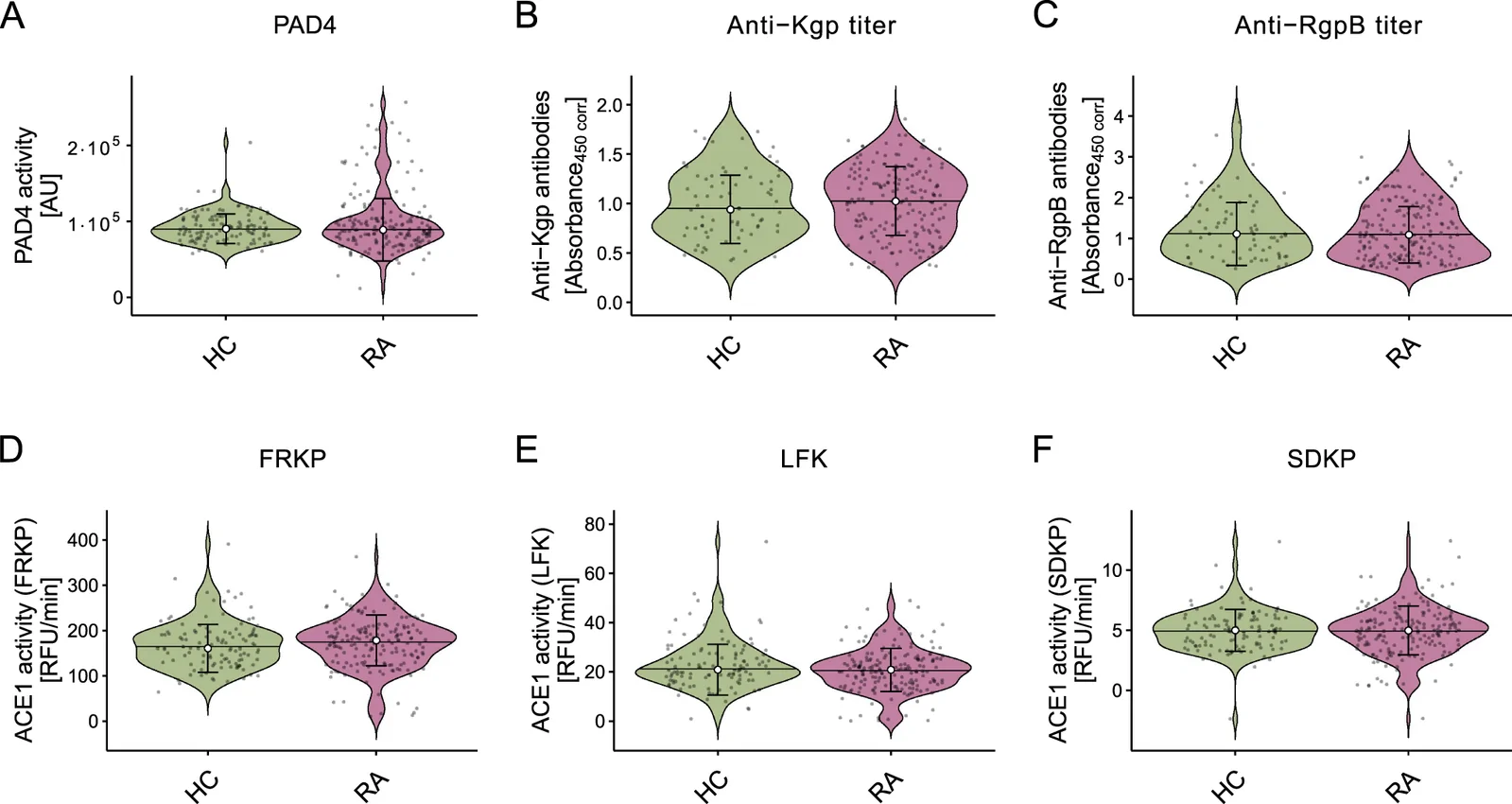
Comparison of selected biochemical markers and antibody levels in individuals stratified by disease status: healthy controls (HC) and patients with rheumatoid arthritis (RA). Violin plots illustrate the distribution of individual data points for each parameter with horizontal lines indicating the group median and error bars representing the standard deviation (SD). The graphs show **A** serum PAD4 enzymatic activity, expressed as arbitrary fluorescence units, **B** IgG serum antibody levels against *P. gingivalis* Kgp, **C** IgG serum antibody levels against *P. gingivalis* RgpB, **D** total ACE1 enzymatic activity, assessed with the fluorogenic substrate FRKP, **E** ACE1 C-domain specific activity assessed with the LFK substrate, **F** ACE1 N-domain specific activity, assessed with the SDKP substrate. HC (**A, D-F**): *n* = 115, HC (**B-C**): *n* = 72, RA (**A-F**): *n* = 177. Asterisks denote statistically significant differences between the compared groups (*p < 0.05, **p < 0.01, ***p < 0.001; ns, not significant)

We began by comparing serum levels of key biochemical and immunological markers between patients with RA and HC. The difference in PAD4 – potentiating activity, Kgp and Rgp antibodies levels representing humoral responses to *P. gingivalis* and ACE1 enzymatic activity across all three substrates did not reach statistical significance between RA patients and healthy controls after covariate adjustment (Fig. 1A-F).

**Fig. 2:**
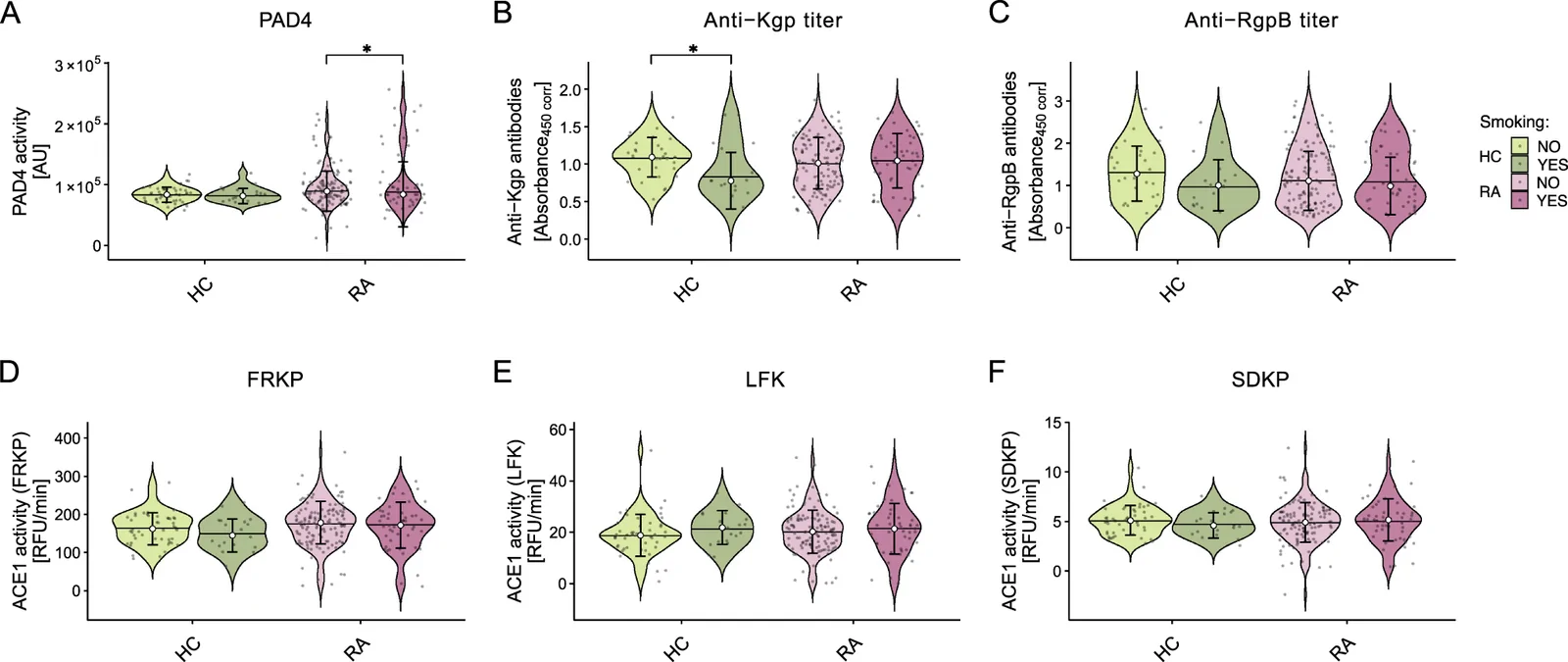
Comparison of selected biochemical markers and antibody levels in smoking (currently or in the past) and non-smoking individuals grouped by disease status. Violin plots illustrate the distribution of individual data points for each parameter, stratified by smoking status, within healthy controls (HC) and patients with rheumatoid arthritis (RA) groups. Horizontal lines indicate the group median and error bars represent the standard deviation (SD). The graphs show (**A**) serum PAD4 enzymatic activity, expressed as arbitrary fluorescence units, (**B**) IgG serum antibody levels against *P. gingivalis* Kgp, (**C**) IgG serum antibody levels against *P. gingivalis* RgpB, (**D**) total ACE1 enzymatic activity, assessed with the fluorogenic substrate FRKP, (**E**) ACE1 C-domain specific activity assessed with the LFK substrate, (**F**) ACE1 N-domain specific activity, assessed with the SDKP substrate. HC no smoking (**A, D-F**): *n* = 48, HC no smoking (**B-C**): *n* = 31, RA no smoking (**A-F**): *n* = 124, HC smoking (**A, D-F**): *n* = 19, HC smoking (**B-C**): *n* = 16, RA smoking (**A-F**): *n* = 49. Asterisks denote statistically significant differences between the compared groups (*p < 0.05, **p < 0.01, ***p < 0.001; ns, not significant)

Further, we investigated the potential influence of smoking status on these biomarkers within the RA group (Fig. 2). In RA, ever-smoking (*n* = 49) was weakly associated with lower PAD4-potentiating activity compared with never-smoking (84,055 [IQR 32,533] vs. 89,230 [IQR 27,182] AU; p_adj_=0.045; Fig. 2A). Anti-Kgp did not differ by smoking within RA, though HC never-smokers had higher anti-Kgp than ever-smokers (1.09 vs. 0.77 AU; p_adj_=0.01). ACE1 activities were not altered by smoking in RA (Fig. 2D–F). These results indicate that while smoking history did not measurably impact anti-*P. gingivalis* antibody levels, or ACE1 activity in this RA cohort, it was associated with decreased serum PAD4-potentiating activity.

#### Subgroup analyses within the RA cohort

**Fig. 3:**
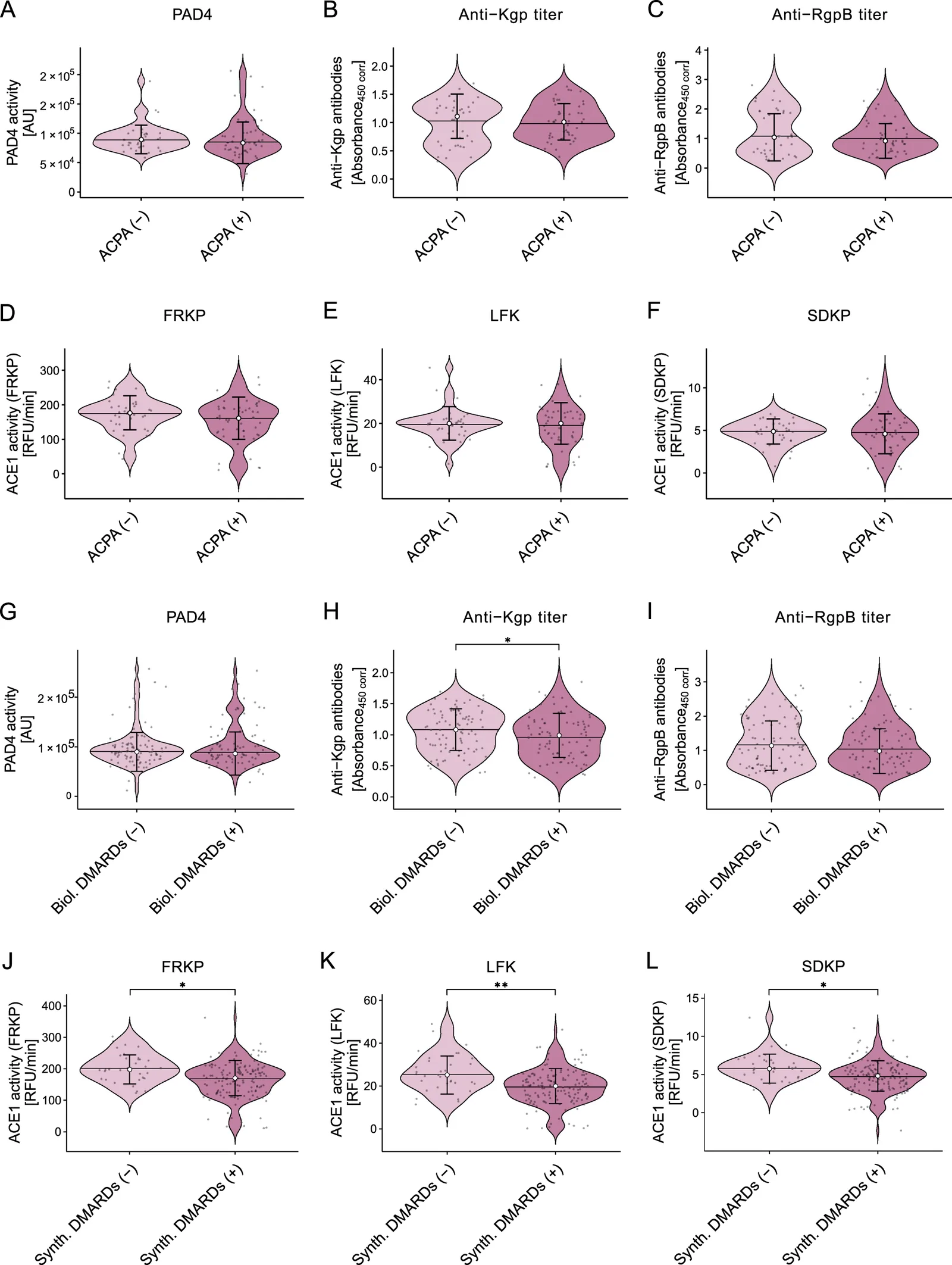
Comparison of selected biochemical markers and antibody levels stratified by ACPA status (**A-F**) and biologic or synthetic DMARDs treatment (**G-L**) within the rheumatoid arthritis (RA) patients group. Violin plots illustrate the distribution of individual data points for each parameter with horizontal lines indicating the group median and error bars representing the standard deviation (SD). The graphs show: (**A, G**) serum PAD4 enzymatic activity, expressed as arbitrary fluorescence units, (**B, H**) IgG serum antibody levels against *P. gingivalis* Kgp, (**C, I**) IgG serum antibody levels against *P. gingivalis* RgpB, (**D, J**) total ACE1 enzymatic activity, assessed with the fluorogenic substrate FRKP, (**E, K**) ACE1 C-domain specific activity assessed with the LFK substrate, (**F, L**) ACE1 N-domain specific activity, assessed with the SDKP substrate. ACPA negative patients: *n* = 36, ACPA positive patients: *n* = 49. Patients on biological DMARDs treatment (not treated: *n* = 92, treated: *n* = 85). Patients on synthetic DMARDs treatment (not treated: *n* = 33, treated: *n* = 144). Asterisks denote statistically significant differences between the compared groups (*p < 0.05, **p < 0.01, ***p < 0.001; ns, not significant)

**ACPA positivity and ACE1 activity:** To explore the relationship between autoantibody status and enzymatic biomarkers, we stratified the RA cohort by ACPA positivity (Fig. 3A-F). Among 85 patients with available ACPA data (49 ACPA-positive and 36 ACPA-negative), no significant differences in PAD4-activity, anti-gingipain antibody levels, or ACE1 activity were observed between ACPA-positive and ACPA-negative RA patients, although FRKP-based ACE1 activity was numerically lower in ACPA-positive group (Fig. 3D).

**Biologic DMARD use and Anti-Kgp antibody levels:** When the cohort was stratified by use of biologic DMARDs (Fig. 3G-I), a significant reduction in anti-Kgp antibody levels was observed among patients receiving biologic therapy. RA patients on biologic DMARDs (*n* = 85) had mean anti-Kgp levels of 0.96 ± 0.36, compared to 1.06 ± 0.34 in those not on biologics (*n* = 92, Fig. 3H), a difference that reached statistical significance (Mann-Whitney U, *p* = 0.019). This pattern was specific to the anti-Kgp response, as neither anti-RgpB levels nor PAD4 activity showed a significant relationship with biologic use.

**Synthetic DMARD use and ACE1 activity**: Patients not currently receiving synthetic DMARDs (*n* = 33) exhibited higher ACE1 activity compared to those on synthetic DMARDs (*n* = 144) across all three substrates (Fig. 3J-L). For the FRKP substrate, mean activity was 204.29 ± 46.32 in non-users versus 164.48 ± 56 in users (*p* = 0.01); for LFK, means were 26.26 ± 8.91 versus 19.51 ± 8.22 (*p* = 0.003); and for SDKP, means were 5.94 ± 1.89 versus 4.68 ± 1.98 (*p* = 0.042). This difference was consistent and statistically robust for all substrates tested. No differences were observed for PAD4 activity or gingipain antibody levels.

**Fig. 4:**
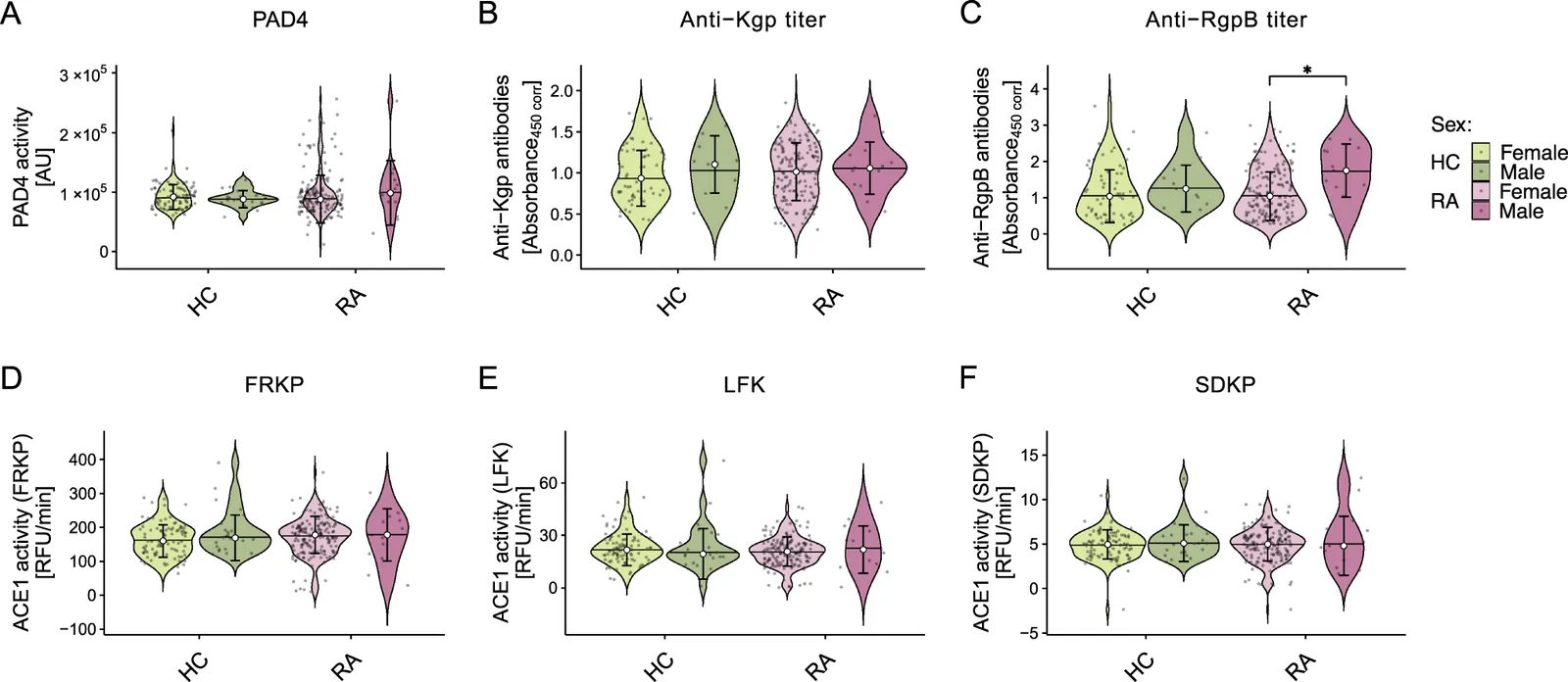
Comparison of selected biochemical markers and antibody levels in female or male individuals grouped by disease status. Violin plots illustrate the distribution of individual data points for each parameter, stratified by sex, within healthy controls (HC) and patients with rheumatoid arthritis (RA) groups. Horizontal lines indicate the group median and error bars represent the standard deviation (SD). The graphs show **A** serum PAD4 enzymatic activity, expressed as arbitrary fluorescence units, **B** IgG serum antibody levels against *P. gingivalis* Kgp, **C** IgG serum antibody levels against *P. gingivalis* RgpB, **D** total ACE1 enzymatic activity, assessed with the fluorogenic substrate FRKP, **E** ACE1 C-domain specific activity assessed with the LFK substrate, **F** ACE1 N-domain specific activity, assessed with the SDKP substrate. HC female (**A, D-F**): *n* = 90, HC female (**B-C**): *n* = 59, RA female (**A-F**): *n* = 164, HC male (**A, D-F**): *n* = 23, HC male (**B-C**): *n* = 11, RA male (**A-F**): *n* = 13. Asterisks denote statistically significant differences between the compared groups (*p < 0.05, **p < 0.01, ***p < 0.001; ns, not significant)

**Sex differences in anti-RgpB antibody levels:** A notable sex difference was observed for anti-RgpB antibody levels (Fig. 4). Male RA patients (*n* = 13) had significantly higher mean anti-RgpB levels than female patients (*n* = 164), with group means of 1.69 ± 0.74 and 1.18 ± 0.67, respectively (Wilcoxon, *p* = 0.015), but due to small subgroup n, this finding should be interpreted cautiously. No sex differences were observed for anti-Kgp, PAD4, or ACE1 activity.

**Fig. 5:**
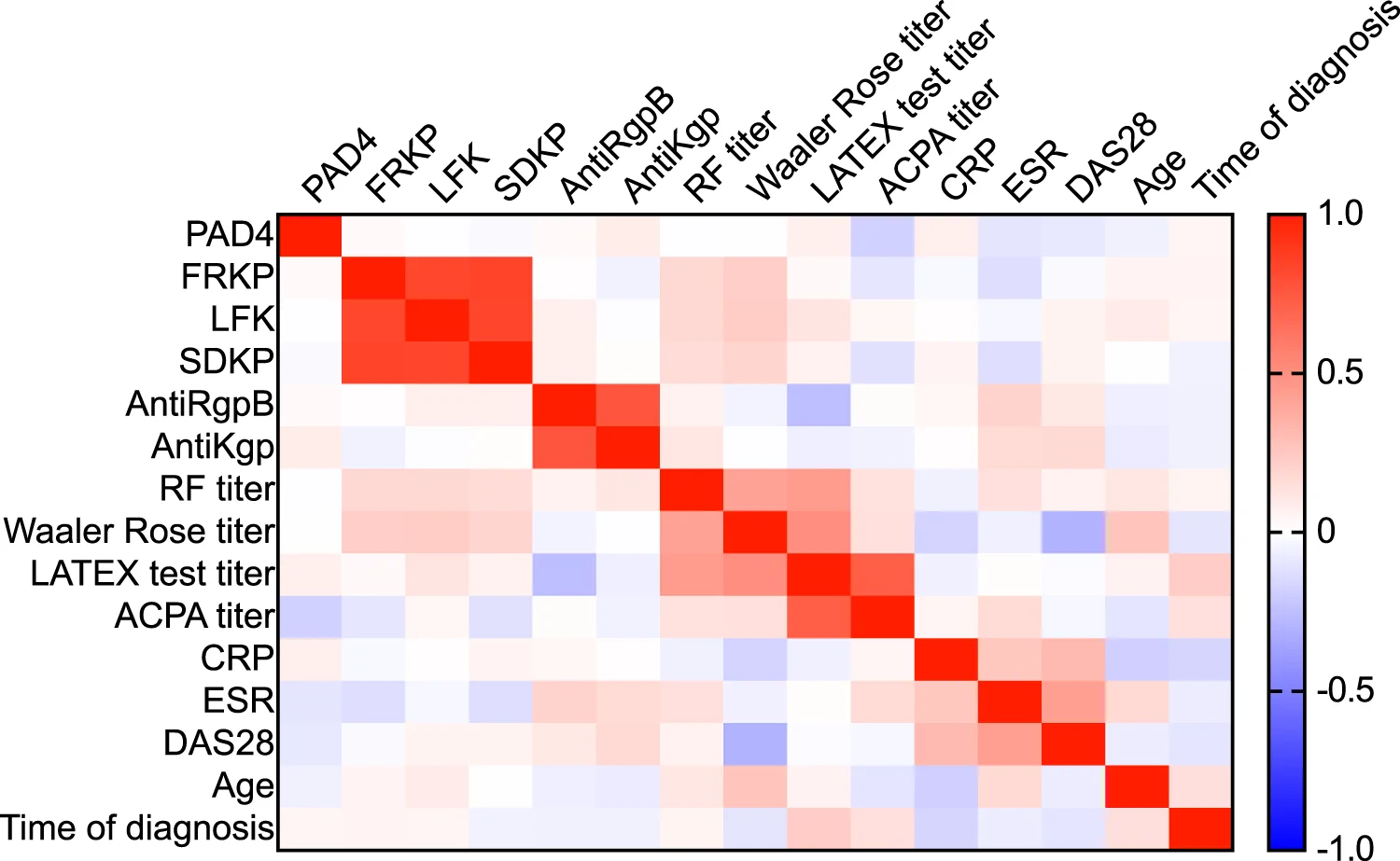
Spearman’s correlation matrix of experimental biomarkers and disease characteristics within the RA group. Color scale represents Spearman’s rank correlation coefficient (Rs), with red representing positive correlations and blue representing negative correlations. Intensity of the color corresponds to correlation strength

**Associations between antibody levels and disease activity or inflammation:** Correlation analysis (Fig. 5) revealed that anti-Kgp antibody levels were weakly but significantly associated with increased disease activity, as measured by DAS28 (Rs = 0.17, *p* = 0.033, *n* = 160), as well as with ESR (Rs = 0.16, *p* = 0.050, *n* = 155), whereas no correlation was observed with CRP. Anti-RgpB was not associated with DAS28, and its initial correlation with ESR was lost after correction for confounders (sex, age, smoking status).

**Multivariate analysis: associations with disease activity**: To assess whether the investigated biomarkers were independently associated with DAS28, we fitted covariate-adjusted linear models including anti-Kgp, anti-RgpB, age, sex, disease duration, smoking, statin use, biologic and synthetic DMARDs, and ACPA. In this analysis, none of the experimental biomarkers showed independent associations with DAS28. Due to small sample size (*n* = 44) with complete data for all variables included in the adjusted model, this analysis was interpreted as exploratory.

**Associations of antibody levels with cardiovascular and metabolic outcomes:** Logistic regression analyses were performed to determine whether anti-Kgp and anti-RgpB antibody levels were related to the presence of CVD (*n* = 8), hypertension (*n* = 92), diabetes (*n* = 28) or periodontal disease, adjusting for age, smoking status and DMARD use. The number of patients included in these models was 176. Neither anti-Kgp nor anti-RgpB levels were significantly associated with the odds of CVD, hypertension, or diabetes. Selected subgroup ROC signals are summarized in Supplementary Tables 1 and 2, but because of the low event counts and multiple comparisons should be interpreted cautiously.

**ACE1 activity and clinical outcomes:** We next examined the role of ACE1 activity in relation to both disease activity and cardiovascular/metabolic comorbidities (Fig. 5; Supplementary Tables 1 and 2). Across all three substrates, ACE1 activity showed no significant correlation with DAS28, CRP, ESR, autoantibody titers, age, or disease duration. In exploratory logistic regression models, ROC analyses showed possible ACE1-related signals for selected prevalent vascular outcomes, particularly for SDKP-based activity after ethnicity adjustment. However, because of the low event counts these findings are presented in Supplementary Tables 1 and 2 and should be considered only as hypothesis-generating observations.

**Ethnicity and biochemical/clinical parameters:** The RA cohort included 95 patients identifying as Mixed/Brown, 65 as White, and 17 as Black. No significant ethnic differences were observed in PAD4 activity, anti-Kgp or anti-RgpB antibody levels, ACE1 activity, disease activity (DAS28), or inflammatory markers CRP or ESR (Kruskal–Wallis tests). Rates of CVD and hypertension were also similar across groups. However, diabetes prevalence was significantly higher among Black (40%) and Mixed/Brown (20%) patients compared to White patients (7%) (*p* = 0.002, Chi-square test). Importantly, ethnicity did not appear to significantly impact any of the primary immunological or enzymatic associations observed in the cohort (Supplementary Fig. 1). Interestingly, PAD4 in patients identifying as Mixed/Brown was associated with higher discrimination for stroke in ROC analyses (AUC = 0.742; *p* = 0.0034). Due to a low number of reported stroke cases, the utility of this biomarker needs to be further validated in a larger cohort.

## Discussion

Using a clinically diverse Brazilian cohort of established RA alongside matched HC, we interrogated three mechanistically distinct biomarker axes: *(i)* the serum potentiation of recombinant PAD4 activity as a proxy for a citrullination-permissive milieu; *(ii)* humoral immunity to *P. gingivalis* (anti-Kgp and anti-RgpB); and *(iii)* domain-resolved ACE1 activity as a readout of vascular–inflammatory tone. At the whole-cohort level none of these measures discriminated RA from HC, but stratified and multivariable analyses identified biologically coherent within-RA associations that may inform future validation efforts. Collectively, our findings highlight anti-Kgp as a therapy-sensitive mucosal biomarker weakly linked to disease activity, raise the hypothesis of a role for N-domain ACE1 activity in vascular remodeling biology, and indicate that serum PAD4 potentiation may be less informative in chronically treated disease.

Anti-Kgp antibody levels showed a weak but statistically significant association with higher DAS28, whereas correlations with ESR did not persist after adjustment. However, anti-Kgp was not independently associated with DAS28 in multivariate analysis. This pattern aligns with prior evidence linking P. gingivalis-specific immune responses to RA activity and inflammation [23, 24]. In contrast, anti-RgpB antibodies showed weaker and less consistent associations, underscoring antigenic specificity within the host response to P. gingivalis. While RgpB-based ELISAs are commonly used and Rgp-targeted inhibitors have shown promise in preclinical inflammation models [25], our results suggest that Kgp-directed humoral responses may more closely reflect clinical disease activity in established RA. This difference in antigenic dominance, systemic exposure or immunopathogenic roles of the two gingipains warrants further comparative investigation.

Anti-Kgp levels were significantly lower among patients receiving biologic DMARDs, suggesting an immunomodulatory effect of biologic therapy on mucosally driven antibody responses. This finding mirrors evidence that anti-TNF and IL-6 inhibitors reduce periodontal disease markers and microbial colonization [26–28]. These observations position anti-Kgp as a therapy-responsive marker rather than a diagnostic discriminator.

ACE1 activities did not differ between RA and HC overall, higher SDKP-based activity (N-domain) showed a trend toward association with CVD, with ethnicity adjustment improving discrimination. The N-domain preferentially hydrolyzes Ac-SDKP, an anti-fibrotic, anti-inflammatory tetrapeptide that limits monocyte/macrophage influx, endothelial-to-mesenchymal transition, and collagen deposition [29–31]. Elevated N-domain activity could therefore deplete Ac-SDKP bioavailability and foster endothelial dysfunction and vascular remodeling – hallmarks of RA-associated CVD – even when CRP and routine activity indices are quiescent. Reports of ACE1 overexpression in inflamed synovium/vasculature [32–34] and links between ACE1 polymorphisms and endothelial dysfunction in RA [35, 36] are consistent with this model. Given the small number of CVD events (*n* = 8), robust incremental-utility analyses are underpowered. ROC analyses were descriptive rather than predictive, therefore the ACE1 (SDKP)-CVD signal should be interpreted cautiously, and ROC findings should be regarded as preliminary observations requiring validation in larger cohorts.

In contrast, we observed no significant differences in PAD4-related enzymatic activity between RA patients and HC. It is critical to emphasize that our assay does not measure endogenous PAD4 levels or activity per se, but rather quantifies the ability of patient serum to modulate the enzymatic activity of recombinant human PAD4 on a synthetic substrate. This approach reflects the net presence of circulating serum factors that can enhance or inhibit PAD4 function – such as calcium, reactive oxygen species, and glycosaminoglycans – rather than PAD4 expression or secretion itself [37–39]. Prior studies have shown elevated PAD4 expression and activity in synovial fluid and neutrophils of early or untreated RA patients [40, 41]. Our findings diverge from those reports, likely reflecting differences in ethnicity, disease stage, treatment exposure, assesed biological sample (here: serum) and readout. The majority of patients in our cohort were on long-term DMARD therapy, including biologic agents that reduce systemic inflammation and may suppress circulating PAD4-potentiating factors. Since our assay relies on an exogenous recombinant PAD4 source, it is particularly suited to capturing the influence of serum milieu on citrullination potential, rather than tissue-specific PAD4 dynamics. Taken together, our data suggest that serum potentiation of PAD4 activity may be most informative in early RA or during active flares, and that its utility as a biomarker in chronically treated populations is limited [18].

Several additional subgroup findings provide insight into immune and enzymatic regulation in RA. ACPA-positive patients exhibited a reduction trend in ACE1 activity toward the FRKP substrate. This may reflect antibody-associated modulation of vascular enzymatic pathways or differences in endothelial homeostasis. Patients not receiving synthetic DMARDs demonstrated significantly higher ACE1 activity across all substrates tested. This suggests that conventional immunosuppressive therapy may influence systemic enzymatic pathways beyond inflammation alone, as was shown previously in case of methotrexate impacting endothelial nitric oxide synthase activity and lipid metabolism – both of which are intimately linked to cardiovascular risk in RA [42, 43].

Within RA, ever-smokers exhibited lower PAD4-potentiating activity, whereas ACE1 activities and anti-*P. gingivalis* antibodies were unaffected. Smoking may differentially modulate the citrullination-permissive serum milieu without materially altering ACE1 profiles or gingipain-directed serology. Although smoking is commonly linked to increased PAD4-mediated citrullination in lungs, this may bear no impact on the serum-associated PAD activity. Lastly, statin use did not correlate with differences in our experimental biomarkers’ levels. As statins have been shown to reduce systemic inflammation, including CRP levels, in RA [44, 45], the absence of biomarker differences in our cohort may reflect limited power, duration of use, or treatment heterogeneity. Sex- and ethnicity-specific observations, including higher anti-RgpB levels in men and increased diabetes prevalence among Black and Mixed/Brown patients, should be interpreted cautiously due to limited subgroup sizes but are consistent with broader reports of immunological and cardiometabolic heterogeneity in RA [46].

Our findings can be placed in the context of previous Rheumatology International reports. González et al. found in a cross-sectional RA cohort that higher disease activity and rheumatic nodules were associated with more severe periodontitis [47], whereas González-Chávez et al. found no correlation between disease activity and periodontitis [48], indicating that this relationship is not consistent across populations. On the cardiovascular side, Fedorchenko et al. reviewed the increased risk of myocardial infarction in rheumatic diseases and the contribution of chronic inflammation, endothelial dysfunction and autoantibodies to vascular injury [49]. Our study extends this work by measuring serum antibodies to *P. gingivalis* gingipains rather than clinical periodontal status, and by assessing domain-resolved ACE1 activity as a specific vascular-remodeling readout that provides a measurable candidate for future cardiovascular-risk studies in RA Table 1.

**Table 1:** Demographics of cohort analysed in the study

		Group		
Characteristic	Overall *N* = 324^a^	HC *N* = 147^a^	RA *N* = 177^a^	*p*-value^b^
AGE	50.74 (13.55)	43.09 (13.05)	56.84 (10.52)	**< 0.001**
No data	6	6	0	
ETHNICITY				**0.003**
Black	51 (16%)	34 (24%)	17 (9.6%)	
Mixed/Brown	154 (48%)	59 (41%)	95 (54%)	
Other	1 (0.3%)	1 (0.7%)	0 (0%)	
White	115 (36%)	50 (35%)	65 (37%)	
No data	3	3	0	
SEX				**0.006**
Female	283 (88%)	119 (82%)	164 (93%)	
Male	39 (12%)	26 (18%)	13 (7.3%)	
No data	2	2	0	
**CO-MORBIDITIES**				
DIABETES	40 (12%)	12 (8.2%)	28 (16%)	0.053
No data	1	0	1	
HYPERTENSION	123 (38%)	31 (21%)	92 (52%)	**< 0.001**
No data	1	0	1	
CARDIOVASCULAR DISEASE	10 (3.1%)	2 (1.4%)	8 (4.5%)	0.2
No data	1	0	1	
CVA (STROKE)	4 (1.2%)	0 (0%)	4 (2.3%)	0.2
No data	1	0	1	
PERIPHERAL ARTERIAL DISEASE	18 (5.6%)	13 (8.8%)	5 (2.9%)	**0.035**
No data	2	1	2	
PERIODONTAL DISEASE	110 (34%)	33 (22%)	77 (44%)	**< 0.001**
No data	3	0	3	
SMOKING (CURRENT OR PAST)	81 (31%)	32 (37%)	49 (28%)	0.2
No data	64	60	4	
SMOKING (CURRENT)	26 (8.1%)	17 (12%)	9 (5.2%)	0.061
No data	4	0	4	
**MEDICATIONS**				
SYNTHETIC DMARDS	144 (44%)	0	144 (81%)	
BIOLOGICAL DMARDS	85 (26%)	0	85 (48%)	
STATINS	63 (20%)	17 (12%)	46 (26%)	**0.002**
No data	1	1	0	

^a^ Mean (SD); n (%); ^b^ Welch Two Sample t-test; Pearson’s Chi-squared test

Key limitations include the cross-sectional design of the study, low cardiovascular event counts, absence of periodontal phenotyping, and lack of data on ACE inhibitors or angiotensin II receptor blockers use and relevant genotypes. As all measurements were obtained at a single time point, this study cannot establish causal relationships or determine whether the investigated biomarkers predict future disease activity or cardiovascular complications. Accordingly, our findings should be interpreted as exploratory associations and not as evidence of prognostic or predictive value. In addition, the healthy controls were younger than the RA patients; although the primary between-group comparisons were not significant and the within-RA models accounted for age, residual age-related confounding cannot be fully excluded. ACPA status was available for only 85 patients, which limits the serological characterisation, and the multivariable model of disease activity was based on 44 complete records, which limits its stability. Many comparisons were performed without formal correction for multiple testing, so nominally significant results in small subgroups may reflect chance. Finally, the single-centre design limits generalisability, and these constraints define the exploratory scope of the work.

In summary, while no single marker distinguished RA from HC, two mechanistically distinct signals emerged within RA: anti-Kgp IgG as a possible therapy-responsive marker of mucosal immune activation, and SDKP-based ACE1 activity as a potential indicator of vascular remodeling biology. These domain-specific serologic and enzymatic measures may capture processes not fully reflected by CRP or DAS28 and warrant prospective validation in cohorts with detailed periodontal assessment and adequately powered cardiovascular outcomes.

### Significance and innovation

• This study uniquely combines PAD4-modulating serum activity, pathogen-directed serology, and domain-specific ACE1 biology in one cross-sectional RA cohort. Within this framework, anti-Kgp antibody levels and domain-resolved ACE1 activity showed weak associations linked to treatment exposure and clinical phenotype, supporting their relevance as candidate biomarkers in established disease.

• The observed patterns reflect mucosal-immune and vascular remodeling axes of RA heterogeneity that extend beyond routine inflammatory indices. Anti-Kgp varied with disease activity and biologic DMARD use, whereas domain-specific ACE1 readouts pointed toward vascular and cardiometabolic features not adequately captured by CRP, ESR, or DAS28.

• These findings support pre-specified prospective studies to determine whether the proposed biomarkers can stratify clinically relevant RA subgroups, particularly in relation to treatment context, phenotype, and vascular risk, while also evaluating their complementary value alongside the standard clinical assessment.

## Supplementary Information

Below is the link to the electronic supplementary material.

Supplementary Material 1

Supplementary Material 2

## Data Availability

Data supporting the study cannot be made available due to ethical considerations. To inquire about access to Risk RA data repository, please contact the biobank manager.
