# Supplementary material for: Anti-gingipain antibodies and ACE1 activity in rheumatoid arthritis: associations with disease activity and cardiovascular comorbidity in a cross-sectional study
Source: Rheumatol Int. 2026 Aug 25;46(9):243. doi: 10.1007/s00296-026-06272-4 (PMC13506669; doi:10.1007/s00296-026-06272-4)
Supplement: Supplementary file 1 [file 296_2026_6272_MOESM1_ESM.docx]

**Supplementary materials**

**Anti-gingipain antibodies and ACE1 activity in rheumatoid arthritis: associations with disease activity and cardiovascular comorbidity in a cross-sectional study**

**Supplementary Table 1. Influence of confounding factors on the predictive value (AUC of ROC curve) for experimental biomarkers in RA patients.**

|  | **AUC (n)**  **No confounders** | **AUC (n)**  **Correction: Sex** | **AUC (n)**  **Correction: Ethnicity** | **AUC (n)**  **Correction: Smoking status** |
| --- | --- | --- | --- | --- |
| **Cardiovascular Disease**  number of diagnoses in RA group: n = 8 | | | | |
| Anti-Kgp antibody | 0.525 (176) | 0.557 (176) | 0.586 (176) | 0.556 (173) |
| Anti-Rgp antibody | 0.592 (176) | 0.612 (176) | 0.629 (176) | 0.614 (173) |
| FRKP (ACE1) | 0.542 (176) | 0.574 (176) | 0.609 (176) | 0.573 (173) |
| LFK (ACE1) | 0.472 (176) | 0.564 (176) | 0.609 (176) | 0.565 (173) |
| SDKP (ACE1) | 0.656 (176) | 0.685 (176) | 0.731 (176) | 0.681 (173) |
| PAD4 | 0.468 (176) | 0.431 (176) | 0.632 (176) | 0.447 (173) |
| **Hypertension**  number of diagnoses in RA group: n = 92 | | | | |
| Anti-Kgp antibody | 0.528 (176) | 0.568 (176) | 0.567 (176) | 0.533 (173) |
| Anti-Rgp antibody | 0.539 (176) | 0.580 (176) | 0.575 (176) | 0.520 (173) |
| FRKP (ACE1) | 0.517 (176) | 0.546 (176) | 0.571 (176) | 0.530 (173) |
| LFK (ACE1) | 0.560 (176) | 0.587 (176) | 0.603 (176) | 0.558 (173) |
| SDKP (ACE1) | 0.543 (176) | 0.570 (176) | 0.597 (176) | 0.545 (173) |
| PAD4 | 0.490 (176) | 0.511 (176) | 0.556 (176) | 0.500 (173) |
| **Peripheral Arterial Disease**  number of diagnoses in RA group: n = 5 | | | | |
| Anti-Kgp antibody | 0.486 (175) | 0.453 (175) | 0.642 (175) | 0.455 (172) |
| Anti-Rgp antibody | 0.428 (175) | 0.413 (175) | 0.667 (175) | 0.598 (172) |
| FRKP (ACE1) | 0.58 (175) | 0.611 (175) | 0.681 (175) | 0.608 (172) |
| LFK (ACE1) | 0.419 (175) | 0.615 (175) | 0.691 (175) | 0.623 (172) |
| SDKP (ACE1) | 0.755 (175) | 0.779 (175) | 0.844 (175) | 0.771 (172) |
| PAD4 | 0.601 (175) | 0.632 (175) | 0.689 (175) | 0.617 (172) |
| **Stroke**  number of diagnoses in RA group: n = 4 | | | | |
| Anti-Kgp antibody | 0.578 (176) | 0.608 (176) | 0.770 (176) | 0.596 (173) |
| Anti-Rgp antibody | 0.719 (176) | 0.744 (176) | 0.852 (176) | 0.732 (173) |
| FRKP (ACE1) | 0.581 (176) | 0.615 (176) | 0.778 (176) | 0.593 (173) |
| LFK (ACE1) | 0.584 (176) | 0.616 (176) | 0.763 (176) | 0.626 (173) |
| SDKP (ACE1) | 0.592 (176) | 0.625 (176) | 0.773 (176) | 0.607 (173) |
| PAD4 | 0.794 (176) | 0.811 (176) | 0.863 (176) | 0.797 (173) |
| **Diabetes**  number of diagnoses in RA group: n = 28 | | | | |
| Anti-Kgp antibody | 0.521 (176) | 0.529 (176) | 0.664 (176) | 0.515 (173) |
| Anti-Rgp antibody | 0.576 (176) | 0.597 (176) | 0.698 (176) | 0.586 (173) |
| FRKP (ACE1) | 0.546 (176) | 0.562 (176) | 0.678 (176) | 0.538 (173) |
| LFK (ACE1) | 0.574 (176) | 0.587 (176) | 0.689 (176) | 0.57 (173) |
| SDKP (ACE1) | 0.524 (176) | 0.543 (176) | 0.672 (176) | 0.523 (173) |
| PAD4 | 0.476 (176) | 0.552 (176) | 0.664 (176) | 0.484 (173) |
| **Periodontal Disease**  number of diagnoses in RA group: n = 77 | | | | |
| Anti-Kgp antibody | 0.477 (174) | 0.473 (174) | 0.531 (174) | 0.556 (171) |
| Anti-Rgp antibody | 0.530 (174) | 0.529 (174) | 0.459 (174) | 0.555 (171) |
| FRKP (ACE1) | 0.548 (174) | 0.551(174) | 0.556 (174) | 0.592 (171) |
| LFK (ACE1) | 0.575 (174) | 0.582 (174) | 0.59 (174) | 0.604 (171) |
| SDKP (ACE1) | 0.623 (174) | 0.629 (174) | 0.625 (174) | 0.654 (171) |
| PAD4 | 0.500 (174) | 0.531 (174) | 0.548 (174) | 0.470 (171) |

AUC – area under ROC curve; n – number of RA patients included in the model.

**Supplementary Table 2. Experimental biomarkers may be promising candidates for comorbidity predictions in RA patient subsets.**

| **Predictor** | **Confounder (subset)** | **AUC *(p)*** | **CI** | **n** |
| --- | --- | --- | --- | --- |
| **Cardiovascular Disease**  number of diagnoses in RA group: n = 8 | | | | |
| FRKP (ACE1) * | Ethnicity (White) | 0.738 *(0.0103)* | 0.556-0.919 | 64 |
| **Hypertension**  number of diagnoses in RA group: n = 92 | | | | |
| Anti-Kgp antibody | Sex (Male subgroup) | 0.875 *(0.0027)* | 0.63-1 | 12 |
| Anti-Kgp antibody | Statins (Yes) | 0.723 *(0.0059)* | 0.564-0.881 | 46 |
| **Peripheral Arterial Disease**  number of diagnoses in RA group: n = 5 | | | | |
| SDKP (ACE1) | None | 0.755 (0.0038) | 0.582-0.928 | 175 |
| SDKP (ACE1) | Sex (Female subgroup) | 0.762 (0.0042) | 0.583-0.941 | 163 |
| SDKP (ACE1) | Smoking status (Non-smokers) | 0.758 *(0.0029)* | 0.588-0.928 | 163 |
| SDKP (ACE1) | Synthethic DMARDs (Yes) | 0.793 *(<0.0001)* | 0.636-0.949 | 143 |
| SDKP (ACE1) | Statins (No) | 0.798 *(0.0037)* | 0.597-0.999 | 129 |
| **Stroke**  number of diagnoses in RA group: n = 4 | | | | |
| LFK (ACE1) | Synthethic DMARDS (Yes) | 0.636 *(0.0203)* | 0.521-0.75 | 144 |
| PAD4 | Ethnicity (Mixed/Brown) | 0.742 *(0.0034)* | 0.580-0.903 | 95 |
| PAD4 | Smoking status (Non-smokers) | 0.786 *(<0.0001)* | 0.657-0.915 | 164 |
| PAD4 | Synthetic DMARDs (Yes) | 0.768 *(<0.0001)* | 0.626-0.91 | 144 |
| **Diabetes**  number of diagnoses in RA group: n = 28 | | | | |
| Anti-Kgp antibody | Ethnicity (White) | 0.717 *(0.011)* | 0.550-0.884 | 64 |
| LFK (ACE1) | Ethnicity (White) | 0.700 *(0.0202)* | 0.531-0.869 | 64 |
| **Periodontal Disease**  number of diagnoses in RA group: n = 77 | | | | |
| Anti-Kgp antibody | Biological DMARDs (Yes) | 0.663 *(0.0115)* | 0.537-0.790 | 82 |
| FRKP (ACE1) * | ACPA status (Positive) | 0.709 *(0.0081)* | 0.554-0.864 | 48 |
| SDKP (ACE1) | None | 0.623 *(0.0037)* | 0.540-0.706 | 174 |
| SDKP (ACE1) | Sex (Female subgroup) | 0.637 *(0.0016)* | 0.552-0.722 | 162 |
| SDKP (ACE1) * | Ethnicity (Mixed/Brown) | 0.700 *(<0.0001)* | 0.594-0.805 | 95 |
| SDKP (ACE1) * | Synthetic DMARDs (Yes) | 0.668 *(<0.0001)* | 0.579-0.756 | 142 |
| SDKP (ACE1) * | Biological DMARDs (Yes) | 0.700 *(0.0015)* | 0.576-0.823 | 82 |
| SDKP (ACE1) | Smoking status (Non-smokers) | 0.635 *(0.002)* | 0.549-0.721 | 162 |
| SDKP (ACE1) | Statins (No) | 0.630 *(0.0083)* | 0.533-0.726 | 129 |
| PAD4 | Sex (Male) | 0.943 *(<0.0001)* | 0.81-1 | 12 |
| PAD4 | Ethnicity (White) | 0.639 *(0.0487)* | 0.501-0.778 | 63 |

AUC – area under curve; CI – confidence interval; n – number of RA patients used in the model.

Predictors and confounders with AUC above 0.6 are shown if p < 0.05.

* Statistical significance for AUC > 0.6 was also observed for LFK.

**Supplementary Fig. 1** Comparison of selected biochemical markers and antibody levels across different ethnic cathegories grouped by disease status. Violin plots illustrate the distribution of individual data points for each parameter, stratified by ethnicity, within healthy controls (HC) and patients with rheumatoid arthritis (RA) groups. Horizontal lines indicate the group median and error bars represent the standard deviation (SD). The graphs show A) serum PAD4 enzymatic activity, expressed as arbitrary fluorescence units, B) IgG serum antibody levels against *P. gingivalis* Kgp, C) IgG serum antibody levels against *P. gingivalis* RgpB, D) total ACE1 enzymatic activity, assessed with the fluorogenic substrate FRKP, E) ACE1 C-domain specific activity assessed with the LFK substrate, F) ACE1 N-domain specific activity, assessed with the SDKP substrate. HC black (A, D-F ): n=24, HC black (B-C): n=17, RA black (A-F): n=17, HC white (A, D-F): n=38, HC white (B-C): n=21, RA white (A-F): n=65, HC mixed/brown (A, D-F): n=49 , HC mixed/brown (B-C): n=32, RA mixed/brown (A-F): n=95.
