# Supplementary material for: Anti-gingipain antibodies and ACE1 activity in rheumatoid arthritis: associations with disease activity and cardiovascular comorbidity in a cross-sectional study
Source: Rheumatol Int. 2026 Aug 25;46(9):243. doi: 10.1007/s00296-026-06272-4 (PMC13506669; doi:10.1007/s00296-026-06272-4)
Supplement: Supplementary file 2 [file 296_2026_6272_MOESM2_ESM.docx]

**Supplementary File S1**

**STROBE Statement — checklist of items for reports of cross-sectional studies**

This study is reported in accordance with the STROBE (Strengthening the Reporting of Observational Studies in Epidemiology) guidance for cross-sectional studies, available through the EQUATOR Network. The right-hand column shows where each item is addressed in the revised manuscript. Entries marked [p. X] indicate that the authors should insert the final page number once the manuscript is paginated for production.

| **Item** | **Recommendation** | **Reported in the revised manuscript** |
| --- | --- | --- |
| **Title and abstract** | | |
| **1 (a)** | Indicate the study's design with a commonly used term in the title or the abstract. | Title and Abstract (the term “cross-sectional” now appears in both). [p. 1,3] |
| **1 (b)** | Provide in the abstract an informative and balanced summary of what was done and what was found. | Abstract, Objective, Methods, Results and Conclusions. [p. 3] |
| **Introduction** | | |
| **2** | Explain the scientific background and rationale for the investigation being reported. | Introduction. [p. 4] |
| **3** | State specific objectives, including any prespecified hypotheses. | Introduction, closing aim paragraph, and Abstract Objective. [p. 3-4] |
| **Methods** | | |
| **4** | Present key elements of study design early in the paper. | Methods, opening paragraph, and Abstract (cross-sectional design). [p. 3-4] |
| **5** | Describe the setting, locations, and relevant dates, including periods of recruitment, exposure, follow-up, and data collection. | Methods, Sample collection and processing, and Ethical Considerations (HUCAM-UFES, Vitória, Brazil; Risk RA repository). [p. 2, 4-5] |
| **6** | Give the eligibility criteria, and the sources and methods of selection of participants. | Methods, participants (RA by ACR/EULAR 2010 criteria; healthy controls). [p. 3-4] |
| **7** | Clearly define all outcomes, exposures, predictors, potential confounders and effect modifiers. Give diagnostic criteria, if applicable. | Methods (DAS28, CRP, ESR, treatments and comorbidities as outcomes and covariates; PAD4-activating potential, domain-resolved ACE1 activity and anti-Kgp/anti-RgpB antibodies as exposures). [p. 3,5-6] |
| **8** | For each variable of interest, give sources of data and details of methods of assessment (measurement). Describe comparability of assessment methods if there is more than one group. | Methods, subsections ACE1 activity, PAD4-activating potential, and Anti-RgpB and anti-Kgp antibodies. The same standardized in-house assays were applied to patients and controls. [p. 5] |
| **9** | Describe any efforts to address potential sources of bias. | Methods, Data analysis (covariate-adjusted models; normalization to a reference serum in each batch) and Discussion, Limitations. [p. 5-6, 10] |
| **10** | Explain how the study size was arrived at. | Methods. The sample reflects the available Risk RA cohort (177 patients and 147 controls). No formal a priori sample-size calculation was performed, as now stated. [p. 3-4] |
| **11** | Explain how quantitative variables were handled in the analyses. If applicable, describe which groupings were chosen and why. | Methods, Data analysis (biomarker values normalized to a reference serum; ACE1 substrate velocities analysed separately; treatment and comorbidity groupings). [p. 5-6] |
| **12 (a)** | Describe all statistical methods, including those used to control for confounding. | Methods, Data analysis (Spearman correlation, linear regression for continuous outcomes, logistic regression for binary outcomes, with covariate adjustment). [p. 5-6] |
| **12 (b)** | Describe any methods used to examine subgroups and interactions. | Methods, Data analysis, and Results (predefined strata such as treatment and sex). [p. 5–8] |
| **12 (c)** | Explain how missing data were addressed. | Methods, Data analysis. Analyses used available cases, and the limited ACPA availability (85 patients) is noted. [p. 4.6] |
| **12 (d)** | If applicable, describe analytical methods taking account of the sampling strategy. | Not applicable. Participants were enrolled from a single cohort without complex sampling. |
| **12 (e)** | Describe any sensitivity analyses. | Methods and Results (false discovery rate analysis, age-overlap analysis, and frequency-matched subset analysis added in revision). [p. 6–8] |
| **Results** | | |
| **13 (a)** | Report the numbers of individuals at each stage of the study. | Results and Table 1 (177 RA patients and 147 controls). [p. 6] |
| **13 (b)** | Give reasons for non-participation at each stage. | Results. [p. 6] |
| **13 (c)** | Consider use of a flow diagram. | Not included. The cohort structure is described in the text and Table 1. |
| **14 (a)** | Give characteristics of study participants and information on exposures and potential confounders. | Results and Table 1 (demographics, disease characteristics, treatments and comorbidities). [p. 6-8] |
| **14 (b)** | Indicate the number of participants with missing data for each variable of interest. | Results and table footnotes (including ACPA available in 85 patients). [p. 4,7] |
| **15** | Report numbers of outcome events or summary measures. | Results (DAS28 distribution and the eight cardiovascular events). [p. 7] |
| **16 (a)** | Give unadjusted estimates and, if applicable, confounder-adjusted estimates and their precision (e.g., 95% confidence intervals). | Results and Tables (unadjusted and covariate-adjusted estimates with confidence intervals). [p. 6–8] |
| **16 (b)** | Report category boundaries when continuous variables were categorized. | Results and Methods where variables were grouped. [p. 5–8] |
| **16 (c)** | If relevant, consider translating estimates of relative risk into absolute risk for a meaningful time period. | Not applicable to this cross-sectional biomarker analysis. |
| **17** | Report other analyses done (e.g., analyses of subgroups and interactions, and sensitivity analyses). | Results (ROC analyses, predefined subgroups, and the sensitivity analyses noted under item 12e). [p. 6-8] |
| **Discussion** | | |
| **18** | Summarise key results with reference to study objectives. | Discussion, opening paragraphs. [p. 8-9] |
| **19** | Discuss limitations, taking into account sources of potential bias or imprecision. Discuss both direction and magnitude of any potential bias. | Discussion, Limitations (expanded in revision). [p. 10] |
| **20** | Give a cautious overall interpretation of results considering objectives, limitations, multiplicity of analyses, results from similar studies, and other relevant evidence. | Discussion. [p. 8–10] |
| **21** | Discuss the generalisability (external validity) of the study results. | Discussion, Limitations (single-centre cohort). [p. 10] |
| **Other information** | | |
| **22** | Give the source of funding and the role of the funders for the present study and, if applicable, for the original study on which the present article is based. | Statements and Declarations, Funding. [p. 2] |

*Reference: von Elm E, Altman DG, Egger M, Pocock SJ, Gøtzsche PC, Vandenbroucke JP; STROBE Initiative. The Strengthening the Reporting of Observational Studies in Epidemiology (STROBE) statement: guidelines for reporting observational studies. Ann Intern Med. 2007;147(8):573-577.*

*The STROBE checklist is distributed under the terms of the Creative Commons Attribution licence and is available at www.strobe-statement.org.*
